# Alpha-lipoic acid could attenuate the effect of chemerin-induced diabetic nephropathy progression

**DOI:** 10.22038/ijbms.2021.50792.11570

**Published:** 2021-08

**Authors:** Hong Zhang, Jiawei Mu, Jinqiu Du, Ying Feng, Wenhui Xu, Mengmeng Bai, Huijuan Zhang

**Affiliations:** 1 Department of Endocrinology, First Affiliated Hospital of Harbin Medical University, Harbin, China

**Keywords:** Alpha-lipoic acid, Chemerin, Diabetic nephropathy, Nuclear factor-kappa-B, P38 mitogen-activated - protein kinases

## Abstract

**Objective(s):**

Chemerin is associated with insulin resistance, obesity, and metabolic syndrome. α-lipoic acid (α-LA) is a potent antioxidant involved in the reduction of diabetic symptoms. This study aimed to investigate the relationship between chemerin and P38 MAPK in the progression of diabetic nephropathy (DN) and examine the effects of α-LA on chemerin-treated human mesangial cells (HMCs).

**Materials and Methods:**

HMCs were transfected with a chemerin-overexpressing plasmid. HMCs were also treated with high-glucose, chemerin, α-LA, PDTC (pyrrolidine dithiocarbamate ammonium, NF-κB p65 inhibitor), and/or SB203580 (P38 MAPK inhibitor). Cell proliferation was tested using the Cell Counting Kit-8 assay. Collagen type IV and laminin were tested by ELISA. Chemerin expression was detected by qRT-PCR. The chemerin receptor was detected by immunohistochemistry. Interleukin-6 (IL-6), tumor necrosis factor-a (TNF-α), nuclear factor-κBp-p65 (NF-κB p-p65), transforming growth factor-β (TGF-β), and p-P38 mitogen-activated protein kinase (p-P38 MAPK) were evaluated by western blot.

**Results:**

High-glucose culture increased the expression of the chemerin receptor. α-LA inhibited HMC proliferation. Chemerin overexpression increased collagen type IV and laminin expression. P38 MAPK signaling was activated by chemerin, resulting in up-regulation of IL-6, TNF-α, NF-κB p-p65, and TGF-β. SB203580, PDTC, and α-LA reversed the effects of chemerin, reducing IL-6, TNF-α, NF-κB p-p65, and TGF-β expression.

**Conclusion:**

Chemerin might be involved in the occurrence and development of DN. α-LA might prevent the effects of chemerin on the progression of DN, possibly via the P38 MAPK pathway.

## Introduction

As one of severely microvascular complications of diabetes (1-3), DN involves the thickening of the capillary basement membrane and microvascular endothelial cell hyperplasia, leading to glomerular sclerosis, thickening of the glomerular basement membrane, mesangial expansion, and extracellular matrix hyperplasia (4). These changes cause a high glomerular filtration rate and proteinuria, leading to chronic renal insufficiency (4).

Chemerin is an adipokine that regulates the differentiation and metabolism of adipose tissue through auto-/paracrine signaling (5). It is secreted as an 18-kDa inactive pro-protein and undergoes extracellular serine protease cleavage of its C-terminal portion to generate the 16-kDa active chemerin, which is present in the plasma, serum, hemofiltrate, and kidney (6). It plays a potential role in controlling local immune responses and inflammation of tissue injury, including chronic inflammation of adipose tissue in obesity (6). In the general population, serum chemerin levels are inversely correlated with renal function (7). 

α-lipoic acid (α-LA) belonging to the B vitamin family is an important metabolic anti-oxidant in the human body (8). It can be used as an antidote for heavy metal poisoning and is regarded a universal anti-oxidant (9). α-LA is regarded as a novel therapeutic agent in kidney diseases (10), and its potential to treat DN is being explored (11). α-LA attenuates the toxic effect of high glucose levels on cells (12). α-LA suppresses neuronal excitability and colonic hypersensitivity in diabetic rats (13). α-LA prevents the atrophy of slow and fast muscles in diabetes (14).

The P38-MAPK signaling pathway is critical in many pathophysiologic processes such as inflammation, oxidative stress, cell cycle, and apoptosis. Higher P38 phosphorylation is observed in diabetic patients, and phosphorylated P38 was identified in accumulating interstitial macrophages and myofibroblasts. With type 1 and type 2 diabetic mice, the level of phosphorylated P38 in the kidney increases (2–6 times). Further assessment of streptozotocin-induced diabetic nephropathy showed that interstitial phosphorylated P38 correlated with interstitial fibrosis (15). Many studies showed that P38-MAPK could be used as a treatment target against impaired glucose metabolism (16, 17) and diabetic complications like myocardial collagen deposition (18). Inhibition of P38-MAPK using dexmedetomidine protects against renal ischemia and reperfusion injury (19). 

Studies have shown that in glomerular endothelial cells, high glucose stimulation can induce expression of chemerin, which can promote activation of cytokines through the p38 MAPK signaling pathway (20), and DN patients as well as mice models show increased levels of chemerin expression, and suppressing ChemR23 (receptor for chemerin) alleviates DN damage (21). There are studies about the effects of chemerin in diabetic complications (22-27), as well as on the effects of α-LA in diabetes (24, 28-50) (of note, most studies of the effects of α-LA are about diabetic neuropathy, not DN), but to our knowledge, there have been no previous studies that investigated the effect of α-LA in chemerin-induced human mesangial cell injury and the underlying mechanisms. Therefore, the purpose of this study was to explore the relationship between chemerin and P38 MAPK in the development of DN using human mesangial cells (HMCs) and to examine the effects of α-LA on chemerin induced HMCs. The innovation of this article is to discuss the protective effect of α-LA on chemerin-induced kidney damage.

## Materials and Methods

***Cell culture***

HMCs were purchased from Central South University (Changsha, China). HMCs were cultured in Dulbecco’s Modified Eagle Medium (DMEM; GIBCO, Carlsbad, CA, USA) (glucose concentration was 1.0 g/l) and supplemented with 10% fetal bovine serum (FBS; Sciencell Research Laboratories, Carlsbad, CA, USA) and 1/100 penicillin-streptomycin at 37 °C in 5% CO_ 2_ in a humid atmosphere. 

***Cell transfection***

About 3×10^5^ HMCs were plated in a six-well plate. When they reached 80% confluency, the cells were transfected with the chemerin plasmid (amino acid 118-609; Gene Chemical Technology Co. Shanghai, China). Chemerin plasmid was structured based on the GV144 carrier. The component sequence is CMV-EGFP-MCS-SV40-Neomycin. Plasmid vector alone using Lipofectamine 2000 was applied as transfection medium (Invitrogen, Carlsbad, CA, USA) according to the manufacturer’s instructions. Forty-eight hours later, transfected HMCs were harvested and used for the subsequent experiments. 

***Cell proliferation assay***

Cell proliferation was measured using the Cell Counting Kit-8 (CCK-8; Dojindo Molecular Technologies, Kimamoto, Japan). HMCs were seeded into 96-well plates, at 3.5×10^3^ cells per well. Twenty-four hours later, cells were treated with high glucose (4.5 g/l) and/or 50, 100, 200, 300, 400, and 500 µmol/l of α-LA (Sigma, St Louis, MO, USA), PDTC (Biomol GmbH, Hamburg, Germany), SB203580 (Biomol GmbH, Hamburg, Germany) (5, 10, 20, 50, 100, 200, and 300 µmol/l of each inhibitor), and 0.01, 0.05, and 0.1 µg/ml of Chemerin (amino acid 118-609; Gene Chemical Technology Co., Shanghai, China). After 24 hr, 10 µl of CCK-8 reagent was added, incubated for 1 hr at 37 °C, and the absorbance (OD) was measured at 450 nm with a spectrophotometric plate reader (Bio-Rad, Hercules, CA, USA).

***Enzyme-linked immunosorbent assay (ELISA)***

This assay was based on the sandwich enzyme immunoassay technique. HMCs were centrifuged at 1000 ×g for 20 min, and the supernatants were used for ELISA. The samples and standard solutions (100 μl per well) were added to the microplate that had been pre-coated with anti-mouse collagen type IV (SEA180Hu) and laminin (LN, SEA082Hu) antibodies and were incubated at 37 °C for 2 hr. Rabbit biotinylated polyclonal antibodies for collagen type IV and LN were added, incubated for 30 min, and washed four times with PBS buffer. HRP-conjugated streptavidin was added to each well, incubated for 30 min at 37 °C, and washed four times with PBS buffer. The Substrate reagent TMB and stop solution were added to each well in sequence. The absorbance was immediately measured at 450 nm using a spectrophotometric plate reader (Bio-Rad, Hercules, CA, USA).

***Quantitative real-time RT-PCR (RT-qPCR)***

Trizol (Invitrogen, Carlsbad, CA, USA) was used to extract total RNA from cells, according to the manufacturer’s instructions. RNA purity and concentrations were assessed by measuring the absorbance at 260 and 280 nm using a Nanodrop ND-1000 spectrophotometer (Thermo Fisher Scientific, Waltham, MA, USA). Total RNA was reverse transcribed into cDNA using PrimeScript RT reagent Kit (TaKaRa, Otsu, Japan). The expression of chemerin was measured by real-time PCR using an ABI 7500 real-time PCR System (Applied Biosystems, Foster City, CA, USA) using the following conditions: 95 °C for 30 sec, 40 cycles of 95 °C for 5 sec , and 60 °C for 40 sec. The expression of the target gene was normalized to that of β-actin using 2^-^^ΔΔ^^CT^. The experiment was conducted three times. The primer sequence was shown in Table 1.

***Immunocytochemistry***

HMCs were grown in normal and high glucose conditions. HMCs were inoculated on sterile glass coverslips and placed into 6-well plates. The cells were treated with 1.0 g/l and 4.5 g/l of glucose for 24 hr and incubated at 37 °C in a humidified CO_2_ incubator until they were 50%–70% confluent. The cells were fixed by incubating in 4% (v/v) paraformaldehyde in PBS for 20 min at room temperature and then washed thrice with PBS. The cells on the coverslips were heated for 10 min at 95 °C in the Antigen Retrieval Buffer (100 mM Tris, 5% (w/v) urea, pH 9.5) and then washed thrice with PBS. The cells were permeabilized in 0.1% Triton X-100 in PBS for 15 min at room temperature and washed thrice in PBS. Then the cells were blocked with normal goat serum for 30 min at 37 °C and then incubated with ChemR23 (1:500, ab64881 Abcam) overnight at 4 °C. After washing with PBS, the sections were incubated with Goat Anti-Rabbit IgG H&L-HRP (1: 1000, ab205718 Abcam) for 30 min at room temperature. The sections were stained with 3,3’- Diaminobenzidine (DAB) and counterstained with hematoxylin. The cells were air-dried and mounted on a clean glass slide using a mounting medium. The coverslips were sealed using nail polish, and the images were captured with 40× objective under a light microscope (TE2000, Nikon, Tokyo, Japan). The number of ChemR23 positive HMCs of 10 randomly selected non-overlapping fields was evaluated. The images were analyzed using Image J (National Institutes of Health, Bethesda, MD, USA).

***Western blot analysis***

HMCs were lysed in RIPA lysis buffer (Beyotime Institute of Biotechnology, Haimen, China). Protein concentration was measured using the Bradford assay. Equal amounts of protein per lane were separated by sodium dodecyl sulfate-polyacrylamide gel electrophoresis (SDS-PAGE, 8%) and then transferred to a nitrocellulose membrane (Millipore Corp., Billerica, MA, USA). Membranes were blocked with 5% non-fat milk for 1 hr at room temperature, then incubated with primary antibodies against NF-κBp-p65 (3031s, Cell Signaling, Danvers, MA, USA), P38-MAPK (9212s, Cell Signaling, Danvers, MA, USA), p-P38 MAPK (9211s, Cell Signaling, Danvers, MA, USA), transforming growth factor-β1 (TGF-β1) (3711s, Cell Signaling, Danvers, MA, USA), GAPDH (KC-5G5, Kangcheng Bio-tech, Shanghai, China), β-actin (WL01372, Wanleibio, China), (all at 1:1000), IL-6 (WL02841, Wanleibio, China, 1:500) and TNF-α (WL01581, Wanleibio, China, 1:500) overnight at 4 °C. Then, the corresponding secondary antibodies were added at room temperature for 1 hr. Protein bands were detected by electrochemiluminescence (ECL). A ChemiDoc™ XRS+ System with Image Lab Software (Bio-Rad, Hercules, CA, USA) was used for densitometric analysis of the protein bands.

***Statistical analysis***

All data were presented as mean ± standard deviation (SD). SPSS 17.0 (IBM, Armonk, NY, USA) was used for statistical analysis, and Student’s t-test (comparisons of two groups) or one-way analysis of variance (ANOVA) (analysis of three groups or more) with Tukey’s *post hoc* test was used for comparison. *P*<0.05 was considered statistically significant.

## Results

***High glucose-induced expression of ChemR23 in HMCs***

**Figure 1:**
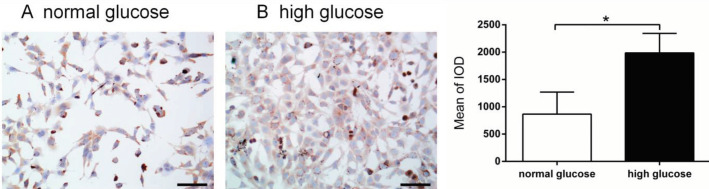
Expression of ChemR23 in human glomerular mesangial cells. (A) HMCs+ glucose (1.0 g/l); (B) HMCs+ glucose (4.5 g/l) treatment for 24 hr. * *P*<0.05 vs glucose (1.0 g/l). The experiments were performed in triplicate. Original magnification, ×200. The scale bar, 100 μm

HMCs were inoculated with different concentrations of glucose for 24 hr. ChemR23 expression was detected by immunocytochemistry. Comparing with the normal control group, the expression of ChemR23 in the high glucose group was significantly increased (P=0.0228, Figure 1). This suggests that ChemR23 may play a vital role in the occurrence and development of diabetic nephropathy.

***Chemerin, P38 MAPK, NF-κBp65, and α-LA are involved in HMC proliferation***

**Figure 2:**
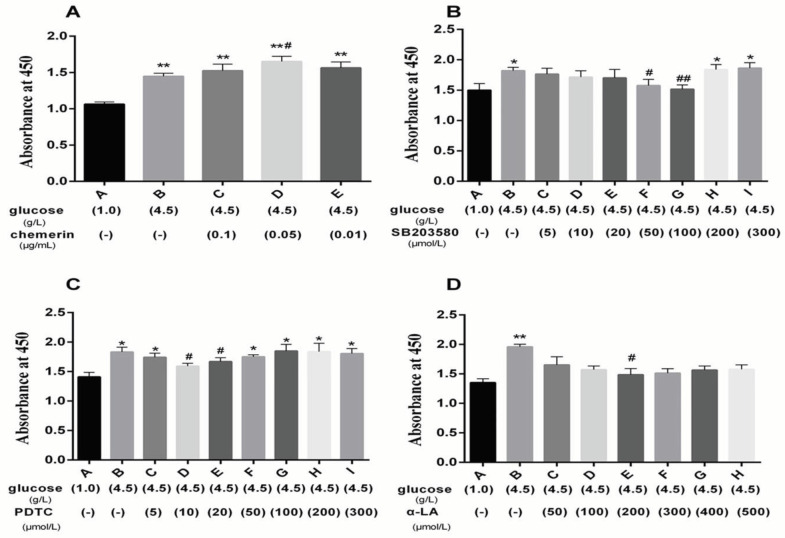
CCK-8 was used to assess cell proliferation of HMCs treated with high glucose and various concentrations of chemerin, SB203580, PDTC, or α-LA. (A) HMCs were treated with chemerin for 24 hr. (B-C) HMCs were treated with SB203580 or PDTC for 1 hr. (D) HMCs were treated with α-LA for 24 hr. Data were derived from four independent experiments. * *P*<0.05, ** *P*<0.01 vs control group. # *P*<0.05, ## *P*<0.01 vs high glucose group. The experiments were performed in triplicate

The effects of chemerin on HMC proliferation were assessed. The results indicated that high glucose enhanced cell proliferation, and chemerin could further promote this effect with 0.05 µg/ml showing the maximum effect (*P*<0.01) (Figure 2A). P38-MAPK inhibitor SB203580 and NF-κB p65 inhibitor PDTC decreased the HMC viability with maximum inhibition at 100 µmol/l and 10 µmol/l, respectively (*P*<0.01) (Figures 2B-C). α-LA decreased HMC proliferation, with the maximum effect being seen at 200 µmol/l (*P*<0.01) (Figure 2D). These results indicate that chemerin, P38-MAPK, NF-κB p65, and α-LA play roles in HMC proliferation.

***Expression of p-P38 MAPK are significantly increased after chemerin treatment***

**Figure 3:**
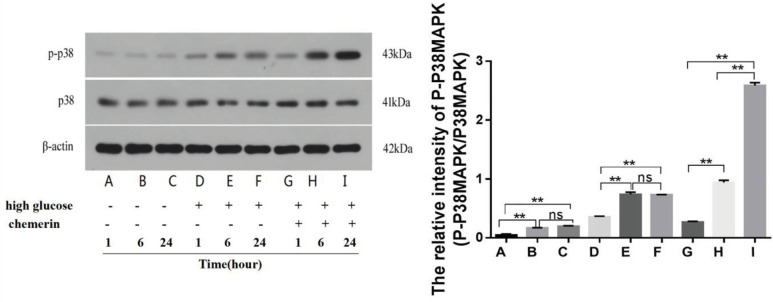
Protein expression of p-p38MAPK was detected by western blot at 1 hr, 6 hr, and 24 hr after chemerin treatment in each group. **P<0.01. The experiments were performed in triplicate

Chemerin (0.05 µg/ml) was used to treat HMCs in conventional culture with different glucose concentrations (1.0 g/l for normal and 4.5 g/l for high glucose) and durations (1, 6, and 24 hr). Compared with 1 hr, the expression of p-P38 MAPK significantly increased after 6 or 24 hr of normal or high glucose or chemerin treatment (*P*<0.01). Compared with 6 hr, the expression of p-P38 MAPK increased significantly after chemerin treatment for 24 hr (*P*=0.0080) (Figure 3). According to these results, we can conclude that chemerin governs the expression of p-P38 MAPK in a time-dependent manner.

***Chemerin increases the expression of collagen type IV and LN***

**Figure 4:**
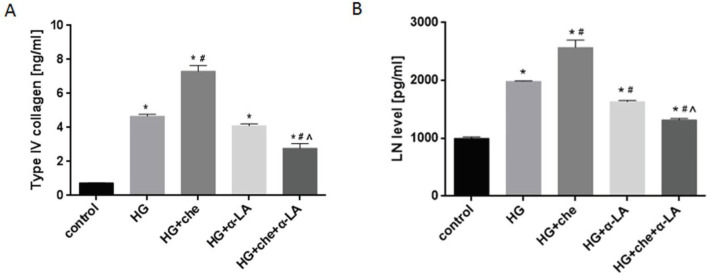
ELISA was used to measure the concentration of (A) type IV collagen and (B) laminin (LN) in cell supernatant. α-LA (200 µM) was used to pretreat the cells for 24 hr, and then chemerin was added and incubated for another 24 hr. *P<0.05 vs control group. #*P*<0.05 vs the high glucose group. ^*P*<0.05 vs high glucose + chemerin group. The experiments were performed in triplicate

**Figure 5:**
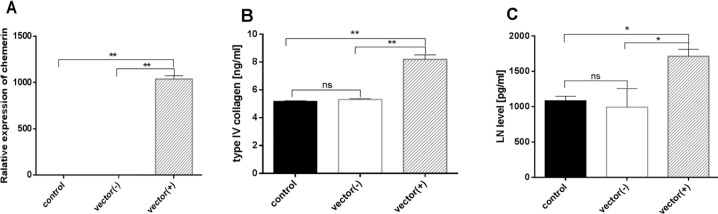
(A) qRT-PCR was used to detect the transfection efficiency of HMC transfected with the chemerin plasmid. (B-C) ELISA was used to measure the concentrations of type IV collagen and laminin (LN) in the cell supernatant. *P<0.05, **P<0.01. The experiments were performed in triplicate

Compared with the normal group, expression of collagen type IV and LN in the cell supernatant showed a remarkable increase in the high glucose group (*P*<0.0001). Collagen Type IV and LN were increased in the chemerin group compared with the high glucose group (*P*=0.0003 and *P*=0.0001). α-LA decreased type IV collagen and LN expression induced by chemerin (*P*<0.0001) (Figures 4A-B). Furthermore, HMCs were transfected with chemerin or vehicle plasmid. As RT-qPCR showed, the relative expression of the chemerin mRNA was 1037 times that of the vehicle group (transfected with only the vector) (*P*=0.0013), while the non-treated group (non-transfected HMCs) was comparable to the vehicle group (*P*=0.0013), (Figure 5A). The expression of collagen type IV and LN was measured by ELISA in the chemerin transfected HMCs, and the results showed that the concentrations of collagen type IV (*P*=0.0091) and LN (*P*=0.043) were significantly increased in the chemerin overexpression group (Figures 5B-C). These results suggest that chemerin aggravates DN by increasing the expression of type IV collagen and LN, whereas α-LA reverses the expression of these proteins.

***The expression of p-P38 MAPK, NF-κB p-p65, and TGF-β was increased after chemerin overexpression***

**Figure 6:**
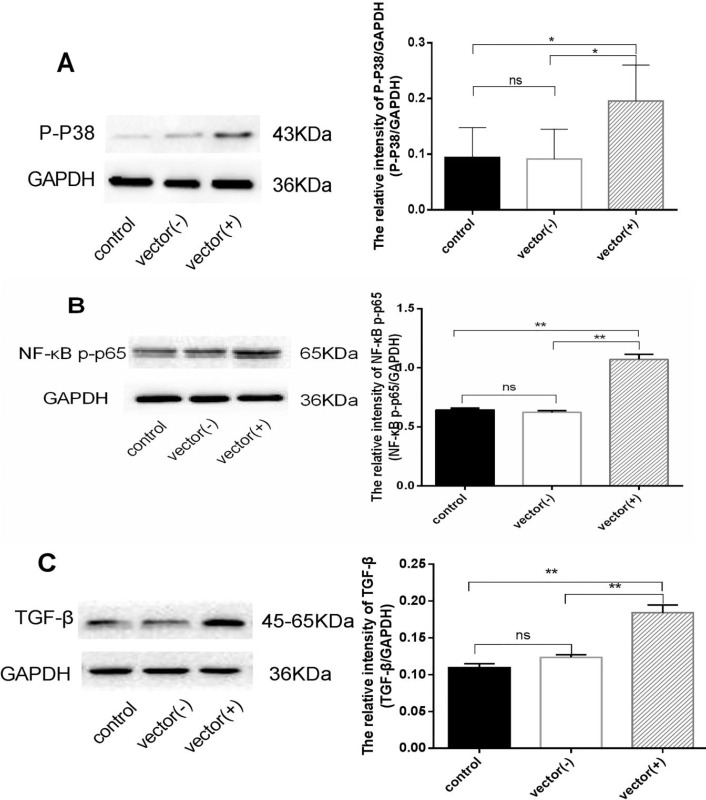
Western blot was used to detect the changes of p-P38 MAPK(A), NF-κB p-p65 (B), and TGF-β (C) expression in HMCs after chemerin plasmid transfection. *P<0.05, **P<0.01. The experiments were performed in triplicate

**Figure 7:**
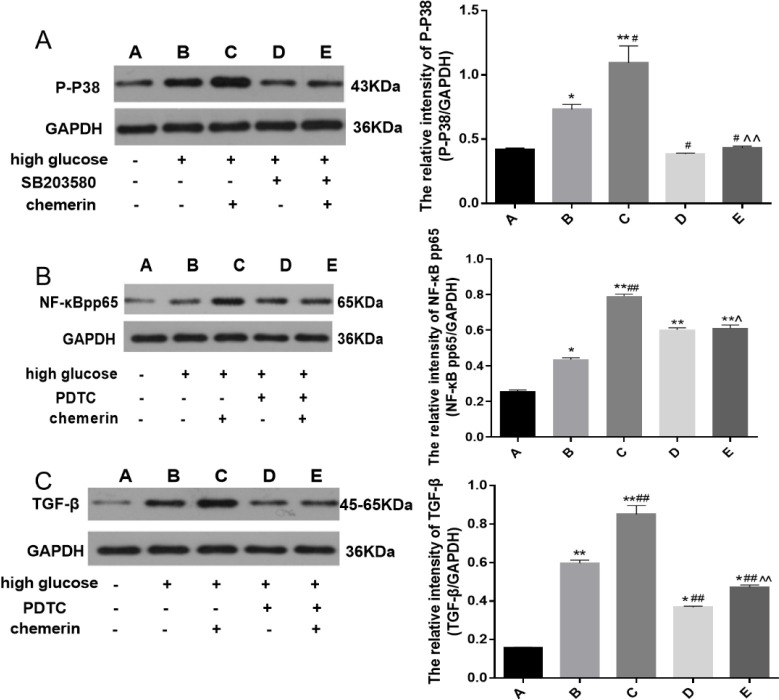
Western blot was used to detect the changes in the expression levels of (A) p-P38 MAPK; (B) NF-κB p-p65; and (C) TGF-β in HMCs after high glucose, SB203580, PDTC, and chemerin treatment. Cells were pretreated with SB203580 or PDTC for 1 hr before chemerin was added and incubated for another 24 hr. *P<0.05, **P<0.01 vs control group. #*P*<0.05,##*P*<0.01 vs high glucose group. ^*P*<0.05, ^^*P*<0.01 vs chemerin group. The experiments were performed in triplicate

Significant increase in the expression of p-P38 MAPK (*P*=0.0488), NF-κB p-p65 (*P*=0.0076), and TGF-β (*P*=0.0096) was seen as compared with the vehicle group, 24 hr post chemerin transfection, suggesting that chemerin influences the expression of these proteins (Figures 6A-C). This phenomenon was further confirmed by chemerin treatment (Figure 7).

***SB203580 blocked the activation of p-P38 MAPK and PDTC abrogated the expression of NF-κB p-p65 and TGF-β induced by chemerin***

Next, we explored the role of chemerin in the expression patterns of p-P38 MAPK, NF-κB p-p65, and TGF-β. Inhibitors namely SB203580 (100 µmol/l) and PDTC (10 µmol/l) were added to HMCs for 1 hr followed by chemerin (0.05 µg/ml). HMCs treated above were cultured for 24 hr. The expression of p-P38 MAPK, NF-κB p-p65, and TGF-β in the high glucose group increased compared with the normal control group (*P*=0.0315, *P*=0.0221, and *P*<0.0001). After treatment with chemerin, levels of all three proteins were higher than in the high glucose group (*P*=0.0002, *P*=0.0001, and *P*<0.0001), while the inhibitors (SB203580 or PDTC) decreased the expression of the proteins (*P*<0.0001,* P*=0.0381, and *P*<0.0001) (Figures 7A-C). These results indicate that chemerin regulates the expression of p-P38 MAPK, NF-κB p-p65, and TGF-β.

***Chemerin increased the expression of IL-6 and TNF-α after SB203580 pretreatment***

**Figure 8.**
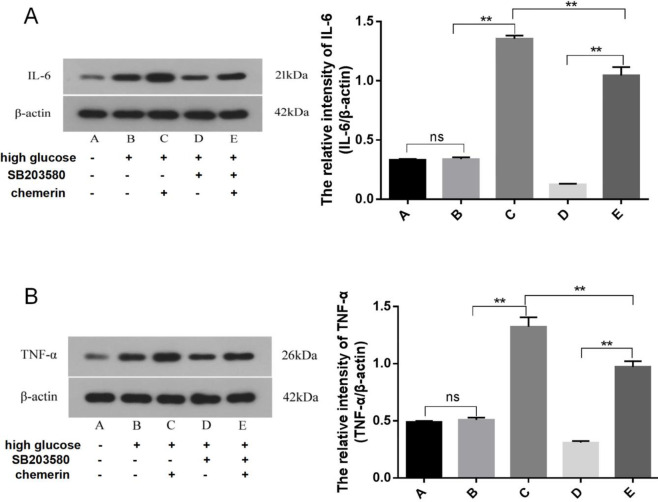
Western blot was used to detect expression of IL-6 (A) and TNF-α (B) in HMCs pretreated with SB203580 for 1 hr followed by addition of chemerin (0.05 µg/ml) and incubation for another 24 hr. **P<0.01. The experiments were performed in triplicate

HMCs were pre-treated with SB203580 (100 µmol/l) for 1 hr and then chemerin (0.05 µg/ml) was added. There were no statistically significant differences in the levels of IL-6 and TNF-α between the normal control group and the high glucose group. Compared with the high glucose group, the expression of IL-6 and TNF increased significantly after treatment with chemerin and high glucose (*P*<0.0001), while SB203580 treatment decreased the expression of IL-6 and TNF-α (*P*=0.0011 and *P*=0.0005). To sum up, chemerin significantly increased the expression of IL-6 and TNF-α (Figure 8).

***α*** ***-*** ***LA decreased the*** ***expression of p-P38 MAPK, NF-κB p-p65, and TGF-β ***

**Figure 9:**
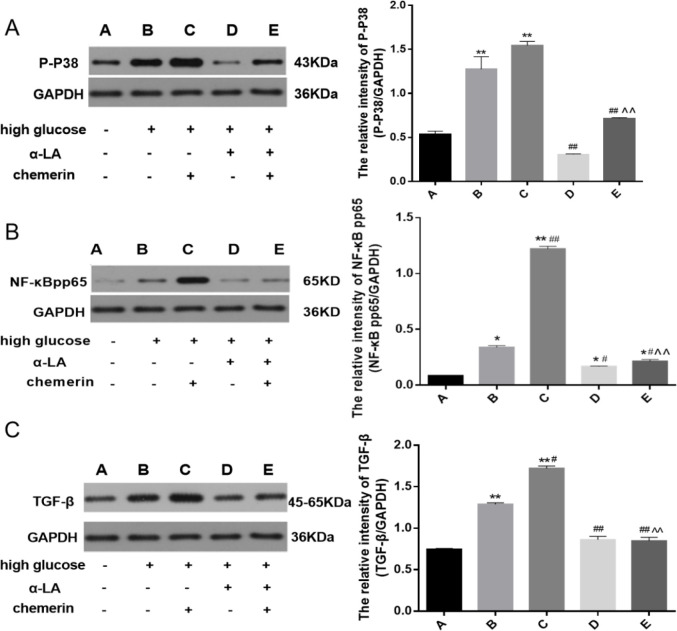
Western blot was used to detect the changes in the expression levels of (A) TGF-β; (B) NF-κB p-p65; and (C) p-P38 MAPK in HMCs after high glucose, α-LA, and chemerin treatment. α-LA (200 µmol/l) was used to pretreat the cells for 24 hr, followed by addition of chemerin (0.05 µg/ml) and incubation for another 24 hr. Data are presented as mean ± SD. A one-way analysis of variance (ANOVA) was used for statistical analysis. *P<0.05, **P<0.01 vs control group. #P<0.05, ##*P*<0.01 vs the high glucose group. ^^*P*<0.01 vs chemerin group. The experiments were performed in triplicate

The expression of p-P38 MAPK, NF-κB p-p65, and TGF-β in the high glucose group was higher than that in the normal control group (*P*=0.0002, *P*=0.0121, and *P*<0.0001). When treated with chemerin and high glucose, the expression of NF-κB p-p65, and TGF-β increased (*P*<0.0001 and *P*=0.0351), while α-LA decreased expression of p-P38 MAPK, NF-κB p-p65, and TGF-β (*P*<0.0001, *P*<0.0001, and *P*=0.0011) (Figure 9). These results suggest that α-LA decreased the expression of p-P38MAPK, NF-κB p-p65, and TGF-β.

## Discussion

There are numerous studies about the involvement of chemerin in diabetic complications (22-27) and the effects of α-LA in diabetic neuropathy (24, 28-47) and DN (48-50), but to our knowledge, there have been no previous studies that investigated the effect of α-LA in chemerin-induced human mesangial cell injury and the underlying mechanisms. The purpose of this study was to explore the relationship between chemerin and P38 MAPK in the development of DN using HMCs and to examine the effects of α-LA on inflammatory cytokines. Our results suggest that chemerin could activate the P38 MAPK signaling pathway resulting in increased expression of p-P38 MAPK, IL-6, TNF-α, NF-κB p-p65, and TGF-β. Expression of type IV collagen and LN was also increased by chemerin overexpression. SB203580, PDTC, and α-LA could reverse the process, leading to a reduced inflammatory reaction. α-LA also inhibited HMC proliferation. 

Chemerin, a novel adipokine, acts via its receptor ChemR23 expressed on macrophages, natural killer cells, immature dendritic cells, endothelial cells, and skeletal muscle cells (51). Serum chemerin is significantly increased in T2DM patients with macroalbuminuria compared with normal and diabetic patients with normoalbuminuria and microalbuminuria; creatinine clearance and serum creatinine are associated with serum chemerin (52). In the diabetic rat model, expression levels of chemerin and ChemR23 in renal tissues are significantly elevated and are positively correlated with TGF-β1, connective tissue growth factor, TNF-α, and intracellular cell adhesion molecule-1 expression (53). TGF-β is involved in renal fibrosis (54-56). The present study showed higher expression of IL-6 and TNF-α, which are representative of inflammatory cytokines in HMCs when treated with chemerin or transfected with chemerin plasmid compared with the normal control group, suggesting that chemerin may potentially play a vital role in the pathology of DN. 

Increased P38 MAPK activity and expression were detected in kidneys of diabetic nephropathy rats (57). Glomerular P38 MAPK activity was increased in early DN, indicating a role in the pathogenesis of early hypertrophy and extracellular matrix accumulation (58). Increased P38 MAPK activity could enhance the expression of TGF-β_1_, a potent inducer of ECM synthesis; enhanced TGF-β_1_ expression has been observed in kidneys from patients and experimental animals with progressive glomerular fibrosis (59, 60). P38 MAPK activation moderates RAGE-induced NF-κB–dependent secretion of proinflammatory cytokines in pancreatic-β cells resulting in accelerated inflammation indicating its significance in the pathophysiological mechanism in diabetes (61). The present study demonstrated elevated expression of p-P38 MAPK, NF-κB p-p65, TGF-β, and ECM proteins such as type IV collagen and LN after chemerin treatment or transfection with chemerin plasmid. When the P38 MAPK inhibitor was added, expression of p-P38 MAPK, IL-6, and TNF-α showed a significant decrease compared with the chemerin group. This suggests that chemerin may stimulate the formation of inflammatory cytokines by activating the P38 MAPK signaling pathway, thereby aggravating the occurrence and development of DN.

There is growing evidence linking chemerin to several diseases. The stimulatory effects of chemerin on fibroblast-like synoviocytes are mediated by activation of P38 MAPK and Akt, while inhibition of P38 MAPK and Akt signaling pathways suppressed chemerin-IL-6 production significantly (62). In human skeletal muscle cells, chemerin release was associated with insulin resistance at the level of lipogenesis and insulin-induced antilipolysis in adipocytes owing to activation of p38 MAPK, NF-κB, and extracellular signal-regulated kinase (ERK)-1/2 by chemerin (63). In human endothelial cells, chemerin activated PI3K/Akt and MAPKs pathways and induced angiogenesis (64). Akin to the previous studies, in the present study, chemerin could promote the expression of p-P38 MAPK and activate the P38 MAPK signaling pathway, leading to increased levels of NF-κB p-p65 and TGF-β. Thus, chemerin might cause renal injury probably by activating the P38 MAPK signaling pathway and releasing inflammatory cytokines

Oxidative stress contributes to the pathogenesis of DN (65). Previous studies show that α-LA can protect renal function in diabetic rodents through its anti-oxidant activity (66) and by regulating glucose oxidation in DN (67); deficiency of lipoic acid synthase increases oxidative stress and accelerates the development of DN (65). Diabetic rats administered with α-LA treatment showed decreased protein levels of plasma malondialdehyde, renal cortical TGF-β_1_, and fibronectin, and urinary protein excretion was positively correlated with renal cortical TGF-β_1_ as well as fibronectin protein levels. These results suggested that α-LA improves proteinuria by reducing the expression of TGF-β_1_ and fibronectin, which in turn were associated with curbing the phosphorylating activation of the P38 MAPK pathway in the renal cortex of the diabetic rats (68). Thus, α-LA protects against DN in various ways. The present study showed that α-LA effectively restored the injury due to diabetes since the expression of p-P38 MAPK, NF-κB p-p65, and TGF-β decreased after supplementing with α-LA compared with the chemerin group. Hence, it is likely that α-LA protects against DN by inhibiting P38MAPK signaling pathway activation.

Chemerin could, at least in part, mediate a relationship between inflammation and cell proliferation. Chemerin is involved in acute and chronic tissue inflammation (6). It participates in the inflammation of the adipose tissue, which is a feature of obesity, and participates in a vicious circle that leads to a progression in the inflammatory state (6). A systemic inflammatory state promotes cell proliferation through a large number of pathways that are too numerous to detail here and are out of the scope of the present study (69-71). Among those mechanisms, the MAPK pathway is involved in cell proliferation in response to inflammation (72, 73). Chemerin, which is elevated in inflammatory states, can activate the MAPK pathway, leading to cell proliferation (20). Nevertheless, the exact molecular steps and interactions among pathways still need to be refined. This will be done in future studies.

**Table 1:** Primer sequences of the chemerin expression after cell transfection with Quantitative Real-time RT-PCR

Genes	Primers	Sequences (5'-3')
Chemerin (Human)	Forward	GCGAACTGTCCAGGGAAGTAGAA
	Reverse	GCGAACTGTCCAGGGAAGTAGAA
β-actin (Human)	Forward	ACTTAGTTGCGTTACACCCTT
	Reverse	GTCACCTTCACCGTTCCA

## Conclusion

In summary, this study provides that chemerin could activate the P38 MAPK signaling pathway inducing inflammatory reactions and NF-κB and TGF-β in HMCs and hence, might be an important pathogenic factor in the development of DN. α-LA, with its ability to improve the injury caused due to diabetes, could prove to be a potential treatment modality for patients with DN. 

## References

[1] Uwaezuoke SN (2017). The role of novel biomarkers in predicting diabetic nephropathy: A review. Int J Nephrol Renovasc Dis.

[2] American Diabetes A (2016). Standards of medical care in diabetes-2016 abridged for primary care providers. Clin Diabetes.

[3] Chamberlain JJ, Rhinehart AS, Shaefer CF Jr, Neuman A (2016). Diagnosis and management of diabetes: synopsis of the 2016 american diabetes association standards of medical care in diabetes. Ann Intern Med.

[4] Bakris GL (2011). Recognition, pathogenesis, and treatment of different stages of nephropathy in patients with type 2 diabetes mellitus. Mayo Clin Proc.

[5] Ma J, Sun F, Wang J, Jiang H, Lu J, Wang X (2018). Effects of aldosterone on chemerin expression and secretion in 3t3-l1 adipocytes. Exp Clin Endocrinol Diabetes.

[6] Weng C, Shen Z, Li X, Jiang W, Peng L, Yuan H (2017). Effects of chemerin/CMKLR1 in obesity-induced hypertension and potential mechanism. Am J Transl Res.

[7] Zylla S, Rettig R, Völzke H, Endlich K, Nauck M, Friedrich N (2018). Serum chemerin levels are inversely associated with renal function in a general population. Clin Endocrinol (Oxf)..

[8] Namazi N, Larijani B, Azadbakht L (2018). Alpha-lipoic acid supplement in obesity treatment: A systematic review and meta-analysis of clinical trials. Clin Nutr.

[9] Saleh HM, El-Sayed YS, Naser SM, Eltahawy AS, Onoda A, Umezawa M (2017). Efficacy of α-lipoic acid against cadmium toxicity on metal ion and oxidative imbalance, and expression of metallothionein and anti-oxidant genes in rabbit brain. Environ Sci Pollut Res Int.

[10] Zhang J, McCullough PA (2016). Lipoic acid in the prevention of acute kidney injury. Nephron.

[11] Gomes MB, Negrato CA (2014). Alpha-lipoic acid as a pleiotropic compound with potential therapeutic use in diabetes and other chronic diseases. Diabetol Metab Syndr.

[12] Dong K, Hao P, Xu S, Liu S, Zhou W, Yue X (2017). Alpha-lipoic acid alleviates high-glucose suppressed osteogenic differentiation of MC3T3-E1 cells via anti-oxidant effect and pi3k/akt signaling pathway. Cell Physiol Biochem.

[13] Sun Y, Yang P-P, Song Z-Y, Feng Y, Hu D-M, Hu J (2017). α-lipoic acid suppresses neuronal excitability and attenuates colonic hypersensitivity to colorectal distention in diabetic rats. J Pain Res.

[14] Jurisic-Erzen D, Starcevic-Klasan G, Ivanac D, Peharec S, Girotto D, Jerkovic R (2018). The effects of alpha-lipoic acid on diabetic myopathy. J Endocrinol Invest.

[15] Adhikary L, Chow F, Nikolic-Paterson DJ, Stambe C, Dowling J, Atkins RC (2004). Abnormal p38 mitogen-activated protein kinase signalling in human and experimental diabetic nephropathy. Diabetologia.

[16] Pang Y, Zhu H, Xu J, Yang L, Liu L, Li J (2017). β-arrestin-2 is involved in irisin induced glucose metabolism in type 2 diabetes via p38 MAPK signaling. Exp Cell Res.

[17] Dhanya R, Arya AD, Nisha P, Jayamurthy P (2017). Quercetin, a lead compound against type 2 diabetes ameliorates glucose uptake via ampk pathway in skeletal muscle cell line. Front Pharmacol.

[18] Guo S, Meng X-W, Yang X-S, Liu X-F, Ou-Yang C-H, Liu C (2018). Curcumin administration suppresses collagen synthesis in the hearts of rats with experimental diabetes. Acta Pharmacol Sin.

[19] Yeda X, Shaoqing L, Yayi H, Bo Z, Huaxin W, Hong C (2017). Dexmedetomidine protects against renal ischemia and reperfusion injury by inhibiting the P38-MAPK/TXNIP signaling activation in streptozotocin induced diabetic rats. Acta Cir Bras.

[20] Zhang X, Wang L, Shang J, Ning LN, Zhao J, Dou Y (2017). Chemerin/ChemR23 promotes high glucose-induced IL-6 and TNF-α expressions in glomerular endothelial cells via p38 MAPK. Chinese Journal of Nephrology.

[21] Shang J, Wang L, Zhang Y, Zhang S, Ning L, Zhao J (2019). Chemerin/ChemR23 axis promotes inflammation of glomerular endothelial cells in diabetic nephropathy. J Cell Mol Med..

[22] El Dayem SM, Battah AA, El Bohy Ael M, El Shehaby A, El Ghaffar EA (2015). Relationship of plasma level of chemerin and vaspin to early atherosclerotic changes and cardiac autonomic neuropathy in adolescent type 1 diabetic patients. J Pediatr Endocrinol Metab.

[23] Bozaoglu K, Bolton K, McMillan J, Zimmet P, Jowett J, Collier G (2007). Chemerin is a novel adipokine associated with obesity and metabolic syndrome. Endocrinology.

[24] Gu P, Wang W, Yao Y, Xu Y, Wang L, Zang P (2019). Increased circulating chemerin in relation to chronic microvascular complications in patients with type 2 diabetes. Int J Endocrinol.

[25] Hu W, Feng P (2011). Elevated serum chemerin concentrations are associated with renal dysfunction in type 2 diabetic patients. Diabetes Res Clin Pract.

[26] Hu W, Yu Q, Zhang J, Liu D (2012). Rosiglitazone ameliorates diabetic nephropathy by reducing the expression of Chemerin and ChemR23 in the kidney of streptozotocin-induced diabetic rats. Inflammation.

[27] Salama FE, Anass QA, Abdelrahman AA, Saeed EB (2016). Chemerin: A biomarker for cardiovascular disease in diabetic chronic kidney disease patients. Saudi J Kidney Dis Transpl.

[28] Bartkoski S, Day M (2016). Alpha-lipoic acid for treatment of diabetic peripheral neuropathy. Am Fam Physician.

[29] Wang X, Lin H, Xu S, Jin Y, Zhang R (2018). Alpha lipoic acid combined with epalrestat: A therapeutic option for patients with diabetic peripheral neuropathy. Drug Des Devel Ther.

[30] Rochette L, Ghibu S, Muresan A, Vergely C (2015). Alpha-lipoic acid: Molecular mechanisms and therapeutic potential in diabetes. Can J Physiol Pharmacol.

[31] Han Y, Wang M, Shen J, Zhang Z, Zhao M, Huang J (2018). Differential efficacy of methylcobalamin and alpha-lipoic acid treatment on symptoms of diabetic peripheral neuropathy. Minerva Endocrinol.

[32] Papanas N, Ziegler D (2014). Efficacy of alpha-lipoic acid in diabetic neuropathy. Expert Opin Pharmacother.

[33] Shay KP, Moreau RF, Smith EJ, Smith AR, Hagen TM (2009). Alpha-lipoic acid as a dietary supplement: molecular mechanisms and therapeutic potential. Biochim Biophys Acta.

[34] Sadeghiyan Galeshkalami N, Abdollahi M, Najafi R, Baeeri M, Jamshidzade A, Falak R (2019). Alpha-lipoic acid and coenzyme Q10 combination ameliorates experimental diabetic neuropathy by modulating oxidative stress and apoptosis. Life Sci.

[35] Chukanova EI, Chukanova AS (2018). [Alpha-lipoic acid in the treatment of diabetic polyneuropathy]. Zh Nevrol Psikhiatr Im S S Korsakova.

[36] Agathos E, Tentolouris A, Eleftheriadou I, Katsaouni P, Nemtzas I, Petrou A (2018). Effect of alpha-lipoic acid on symptoms and quality of life in patients with painful diabetic neuropathy. J Int Med Res.

[37] Cakici N, Fakkel TM, van Neck JW, Verhagen AP, Coert JH (2016). Systematic review of treatments for diabetic peripheral neuropathy. Diabet Med.

[38] Varkonyi T, Korei A, Putz Z, Martos T, Keresztes K, Lengyel C (2017). Advances in the management of diabetic neuropathy. Minerva Med.

[39] Salehi B, Berkay Yilmaz Y, Antika G, Boyunegmez Tumer T, Fawzi Mahomoodally M, Lobine D (2019). Insights on the Use of alpha-Lipoic Acid for Therapeutic Purposes. Biomolecules.

[40] Ibrahimpasic K (2013). Alpha lipoic acid and glycaemic control in diabetic neuropathies at type 2 diabetes treatment. Med Arch.

[41] Gomes MB, Negrato CA (2014). Alpha-lipoic acid as a pleiotropic compound with potential therapeutic use in diabetes and other chronic diseases. Diabetol Metab Syndr.

[42] Nguyen N, Takemoto JK (2018). a case for alpha-lipoic acid as an alternative treatment for diabetic polyneuropathy. J Pharm Pharm Sci.

[43] Snyder MJ, Gibbs LM, Lindsay TJ (2016). Treating painful diabetic peripheral neuropathy: an update. Am Fam Physician..

[44] Vallianou N, Evangelopoulos A, Koutalas P (2009). Alpha-lipoic acid and diabetic neuropathy. Rev Diabet Stud.

[45] Seyit DA, Degirmenci E, Oguzhanoglu A (2016). Evaluation of electrophysiological effects of melatonin and alpha lipoic acid in rats with streptozotocine induced diabetic neuropathy. Exp Clin Endocrinol Diabetes.

[46] Won JC, Kwon HS, Moon SS, Chun SW, Kim CH, Park IB (2020). Gamma-linolenic acid versus alpha-lipoic acid for treating painful diabetic neuropathy in adults: A 12-week, double-placebo, randomized, noninferiority trial. Diabetes Metab J.

[47] Ziegler D, Low PA, Litchy WJ, Boulton AJ, Vinik AI, Freeman R (2011). Efficacy and safety of anti-oxidant treatment with alpha-lipoic acid over 4 years in diabetic polyneuropathy: the NATHAN 1 trial. Diabetes Care.

[48] Feng B, Yan XF, Xue JL, Xu L, Wang H (2013). The protective effects of alpha-lipoic acid on kidneys in type 2 diabetic Goto-Kakisaki rats via reducing oxidative stress. Int J Mol Sci.

[49] Yi X, Xu L, Hiller S, Kim HS, Nickeleit V, James LR (2012). Reduced expression of lipoic acid synthase accelerates diabetic nephropathy. J Am Soc Nephrol.

[50] Xu L, Hiller S, Simington S, Nickeleit V, Maeda N, James LR (2016). Influence of different levels of lipoic acid synthase gene expression on diabetic nephropathy. PLoS One.

[51] Zabel BA, Silverio AM, Butcher EC (2005). Chemokine-like receptor 1 expression and chemerin-directed chemotaxis distinguish plasmacytoid from myeloid dendritic cells in human blood. J Immunol.

[52] Hu W, Feng P (2011). Elevated serum chemerin concentrations are associated with renal dysfunction in type 2 diabetic patients. Diabetes Res Clin Pract.

[53] Hu W, Yu Q, Zhang J, Liu D (2012). Rosiglitazone ameliorates diabetic nephropathy by reducing the expression of Chemerin and ChemR23 in the kidney of streptozotocin-induced diabetic rats. Inflammation.

[54] Wang Y, Liu L, Peng W, Liu H, Liang L, Zhang X (2019). Ski-related novel protein suppresses the development of diabetic nephropathy by modulating transforming growth factor-beta signaling and microRNA-21 expression. J Cell Physiol.

[55] Ding H, Xu Y, Jiang N (2020). Upregulation of miR-101a suppresses chronic renal fibrosis by regulating KDM3A via blockade of the YAP-TGF-beta-smad signaling pathway. Mol Ther Nucleic Acids.

[56] He X, Cheng R, Huang C, Takahashi Y, Yang Y, Benyajati S (2020). A novel role of LRP5 in tubulointerstitial fibrosis through activating TGF-beta/Smad signaling. Signal Transduct Target Ther.

[57] Komers R, Lindsley JN, Oyama TT, Cohen DM, Anderson S (2007). Renal p38 MAP kinase activity in experimental diabetes. Lab Invest.

[58] Kang SW, Adler SG, Lapage J, Natarajan R (2001). p38 MAPK and MAPK kinase 3/6 mRNA and activities are increased in early diabetic glomeruli. Kidney Int.

[59] Kim SI, Kwak JH, Zachariah M, He Y, Wang L, Choi ME (2007). TGF-beta-activated kinase 1 and TAK1-binding protein 1 cooperate to mediate TGF-beta1-induced MKK3-p38 MAPK activation and stimulation of type I collagen. Am J Physiol Renal Physiol.

[60] Hills CE, Squires PE (2010). TGF-beta1-induced epithelial-to-mesenchymal transition and therapeutic intervention in diabetic nephropathy. Am J Nephrol.

[61] Yeh CH, Sturgis L, Haidacher J, Zhang XN, Sherwood SJ, Bjercke RJ (2001). Requirement for p38 and p44/p42 mitogen-activated protein kinases in RAGE-mediated nuclear factor-kappaB transcriptional activation and cytokine secretion. Diabetes.

[62] Kaneko K, Miyabe Y, Takayasu A, Fukuda S, Miyabe C, Ebisawa M (2011). Chemerin activates fibroblast-like synoviocytes in patients with rheumatoid arthritis. Arthritis Res Ther.

[63] Sell H, Laurencikiene J, Taube A, Eckardt K, Cramer A, Horrighs A (2009). Chemerin is a novel adipocyte-derived factor inducing insulin resistance in primary human skeletal muscle cells. Diabetes.

[64] Kaur J, Adya R, Tan BK, Chen J, Randeva HS (2010). Identification of chemerin receptor (ChemR23) in human endothelial cells: Chemerin-induced endothelial angiogenesis. Biochem Biophys Res Commun.

[65] Yi X, Xu L, Hiller S, Kim H-S, Nickeleit V, James LR (2012). Reduced expression of lipoic acid synthase accelerates diabetic nephropathy. J Am Soc Nephrol.

[66] Feng B, Yan X-F, Xue J-L, Xu L, Wang H (2013). The protective effects of α-lipoic acid on kidneys in type 2 diabetic Goto-Kakisaki rats via reducing oxidative stress. Int J Mol Sci.

[67] Yi X, Nickeleit V, James LR, Maeda N (2011). α-Lipoic acid protects diabetic apolipoprotein E-deficient mice from nephropathy. J Diabetes Complications.

[68] Lee SJ, Kang JG, Ryu OH, Kim CS, Ihm S-H, Choi MG (2009). Effects of alpha-lipoic acid on transforming growth factor beta1-p38 mitogen-activated protein kinase-fibronectin pathway in diabetic nephropathy. Metabolism..

[69] Grivennikov SI, Greten FR, Karin M (2010). Immunity, inflammation, and cancer. Cell.

[70] Kim J, Bhattacharjee R, Dayyat E, Snow AB, Kheirandish-Gozal L, Goldman JL (2009). Increased cellular proliferation and inflammatory cytokines in tonsils derived from children with obstructive sleep apnea. Pediatr Res.

[71] Chen L, Deng H, Cui H, Fang J, Zuo Z, Deng J (2018). Inflammatory responses and inflammation-associated diseases in organs. Oncotarget.

[72] Moens U, Kostenko S, Sveinbjornsson B (2013). The role of mitogen-activated protein kinase-activated protein kinases (mapkapks) in inflammation. Genes.

[73] Lu N, Malemud CJ (2019). Extracellular signal-regulated kinase: A regulator of cell growth, inflammation, chondrocyte and bone cell receptor-mediated gene expression. Int J Mol Sci.

